# Documenting response to COVID-individual and systems successes and challenges: a longitudinal qualitative study

**DOI:** 10.1186/s12913-022-08053-8

**Published:** 2022-05-16

**Authors:** Natasha Shaukat, Daniyal Mansoor Ali, Rubina Barolia, Butool Hisam, Sheza Hassan, Badar Afzal, Abdus Salam Khan, Meher Angez, Junaid Razzak

**Affiliations:** 1 Department of Community Health Sciences, Aga Khan University, Stadium Road, P.O. Box-3500, Karachi, 74800 Pakistan; 2 Centre of Excellence for Trauma and Emergencies, Aga Khan University, Karachi, Pakistan; 3 School of Nursing and Midwifery, Aga Khan University, Karachi, Pakistan; 4 Aga Khan University Medical College, Karachi, Pakistan; 5 Department of Emergency Medicine, Aga Khan University, Karachi, Pakistan; 6 Department of Emergency Medicine, Shifa International Hospital, Islamabad, Pakistan; 7 Department of Emergency Medicine, Weill Cornell Medical Center, Newyork, USA

**Keywords:** COVID-19, Frontline healthcare workers, Individual-level challenges, Health system-level challenges, Hope for the future, Prospective longitudinal qualitative study

## Abstract

**Background:**

This study aimed to document the evolution of perceptions of frontline healthcare workers (FHCW) regarding their well-being and the quality of health systems' response to the COVID-19 pandemic over four months in Pakistan.

**Methods:**

We conducted this prospective longitudinal qualitative study during the four months (June–September 2020) coinciding with the peak and trough of the first wave of Pakistan's COVID-19 pandemic. We approached frontline healthcare workers (physicians and nurses) working in emergency departments (ED) in two hospitals using the WhatsApp group of the Pakistan Society of Emergency Physicians (PSEM). Participants were asked to self-record their perception of their wellness and their level of satisfaction with the quality of their hospitals' response to the pandemic. We transcribed, translated, and analysed manually using MAXQDA 2020 software and conducted the thematic analysis to identify themes and sub-themes.

**Results:**

We invited approximately 200 FHCWs associated with PSEM to participate in the study. Of the 61 who agreed to participate, 27 completed the study. A total of 149 audio recordings were received and transcribed. Three themes and eight sub-themes have emerged from the data. The themes were individual-level challenges, health system-level challenges, and hope for the future. Sub-themes for individual-level challenges were: fear of getting or transmitting infection, feeling demotivated and unappreciated, disappointment due to people’s lack of compliance with COVID-19 protocols, physical exhaustion, and fatigue. For the healthcare system, sub-themes were: Infrastructure, logistics, management, and communications response of the hospital/healthcare system and financial stressors. For sub-themes under hope for the future were the improved disease knowledge and vaccine development. The overall perceptions and experiences of FHCWs evolved from fear, grief, and negativity to hope and positivity as the curve of COVID-19 went down.

**Conclusion:**

This study shows that the individuals and systems were not prepared to deal with the challenges of the COVID-19 pandemic. The findings highlight the challenges faced by individuals and health systems during the wake of the Covid-19 pandemic. The healthcare workers were emotionally and physically taxed, while the health systems were overwhelmed by COVID-19. The overall perceptions of FHCWs evolved with time and became negative to positive as the curve of COVID-19 went down during the first wave of COVID-19 in Pakistan.

## Background

The world has been battling the COVID-19 pandemic since December 2019 [1, 2]. As of May 26, 2021, the global pandemic has infected 164 million people worldwide while causing 3.4 million deaths [3–5]. Pakistan confirmed its first COVID-19 case on February 26, 2020, with over 0.9 million confirmed cases and 21,000 deaths by May 26, 2021 [6].

COVID-19 pressurized and crippled healthcare systems across the world [7]. It exposed gaps in public health response and pandemic preparedness even in previously considered high-performing countries [8, 9]. Healthcare services worldwide struggled to respond to the evolving crisis with the Frontline Healthcare Workers (FHCWs) battling the pandemic at tremendous personal risk, often with limited resources [10–13]. FHCWs wellness encompasses physical, mental, and spiritual health and depends on several factors, including stress and burnout [14, 15]. Stressors include day-to-day hospital issues, long working hours, stressful shifts and heavy workload, staff shortages, Personal Protective Equipment (PPE) and supplies shortages, and personal and family pressures [16]. FHCWs suffer psychological distress, anxiety, increased stress, insomnia, depressive symptoms, anger, fear, and post-traumatic stress disorders [16–21]. FHCWS are facing tremendous challenges at work due to the COVID-19 pandemic. Despite these difficulties, the FHCWS and systems continue to adapt to cope with the COVID-19 pandemic “the new normal," i.e., strained hospital capacities, delayed ongoing care, disrupted supply chain, specifically designated hospital areas for Covid-19 screening and treatment of cases, significant adjustment of staff schedules, role and workloads, and a heavy toll on physical, mental, emotional, financial on the healthcare workforce [22–24].

As the pandemic prevails, the prolonged crisis response will lead to further adversities and long-lasting disruption of the overall well-being of FHCWs [15]. Much focus needs to be placed to avoid this helpless situation.

Given the evolution and varying nature of the pandemic, we anticipate the stressors, challenges, and issues to change with time, and data identifying FHCW perceptions may not capture the evolution of the perception over time. Moreover, few similar studies from high-income countries have examined the system and FHCWs challenges faced on a day-to-day basis, their coping mechanisms, and the impact on the overall well-being of FHCWs. For example, a recent longitudinal study of American emergency physicians demonstrated that stress levels decreased with time [25]. On the contrary, a Japanese longitudinal study showed persistent high levels of stress amongst HCWs [26]. To the best of our knowledge, no longitudinal qualitative studies have been conducted during the life of a pandemic in the low- and middle-income countries (LMICs).

This global pandemic is occurring at a time of immense technological advancements. The paradigm shift towards digital health solutions in COVID times is revolutionary [27]. Digital tools can be handy in supporting research in these unprecedented times. The extensive use of smartphones and instant messaging such as (WhatsApp) has become a global phenomenon. WhatsApp is an easy-to-use platform for capturing and generating qualitative data [28]. This means of communication provides several options to participants for self-expression (written, audio, video). They can communicate in real-time as well as asynchronously [27].

We conducted this study to document the evolution of perceptions of frontline healthcare workers (FHCW) regarding their well-being and the quality of health systems' response to the COVID-19 pandemic over four months in Pakistan.

## Methods

### Study setting

Pakistan is the sixth most populous country globally, with over 212 million people. Pakistan has a weak economy and a struggling healthcare system (low health expenditure, i.e., 1.2% of the GDP, poor healthcare infrastructure in rural and urban slum areas, chronic shortages of supplies, dysfunctional medical equipment, and lack of health workforce) [23, 29]. Therefore, Pakistan faced significant challenges in dealing with COVID-19. We conducted the study at two private tertiary care hospitals in Pakistan through the Pakistan Society of Emergency Medicine (PSEM) platform. PSEM is a non-profit professional medical platform representing professionals working in Emergency Medicine. It aims to develop and promote the field of emergency medicine in Pakistan.

### Study design

We conducted a prospective longitudinal qualitative study during the first wave of the COVID-19 pandemic in Pakistan for four months (June–September 2020). During this period, Pakistan experienced the peak of the first wave, followed by the flattening of the curve and ease of lockdown interventions.

### Study population

The study population comprised physicians and nurses involved in clinical service delivery in the Emergency Departments (ED) of Pakistan's two private sector tertiary care hospitals. The ED physicians and nurses in these two hospitals receive the most critically ill and sick patients. As front-liners, the study participants were directly responsible for patients' initial resuscitation and stabilization, initiating diagnosis, and initial management of acute patients.

### Eligibility criteria

We included only those Emergency Medicine physicians and nurses directly involved in taking care of COVID-19 patients in the study.

### Sample size

We invited all (approximately 200) physicians and nurses associated with the Pakistan Society of Emergency Medicine to participate in the study. Initially, 61 people were enrolled in the study; however, 27 remained till the end of the study. We sent weekly reminders to the study participants to send audio recordings—those participants who did not send the audio recording after three reminders were considered to withdraw from the study.

### Sampling technique

We used a convenience sampling technique to select the healthcare workers. We approached the participants through the WhatsApp group of PSEM. A google consent form was shared to invite and obtain consent. Those participants who consented to participate throughout the length of the study were enrolled.

### Data collection protocol

We utilized two methods of data collection: self-audio recordings by participants on WhatsApp and telephonic interviews. The participants were approached through the WhatsApp group of PSEM and emails of individuals. We sent the study guide via WhatsApp to the participants to self-record the answers. The study guide was followed by a detailed WhatsApp text message & audio recording to invite and welcome the participants, introduce the study, and guide them about the data collection process. The message covered the study purpose, what, when, and how to record the audio message and send the audio. We shared a WhatsApp number and requested the participants to self-record short audio messages (four to five minutes) on their mobile phones after every shift's end and send them voice notes/audio recordings via WhatsApp. To ensure the participants kept sending the audio recordings, we sent individual reminders on WhatsApp. Initially, the participants sent two to three audio recordings per week (depending on the number of shifts), with some sending them once a week. Once a week, we telephonically interviewed those participants who could not send audios. Trained researchers (NS and DMA) experienced in qualitative research conducted telephonic interviews in English and Urdu. Overall, we conducted 38 telephonic interviews.

### Study guide

A study guide was prepared and consisted of semi-structured questions. In addition, participants were asked to record audios by answering these open-ended questions:

1. How was your day today? Please describe how are you feeling physically and emotionally today?
2. How was your departments' response to COVID-19 today? What went well? What could have been better?
3. What are you most worried about today?
4. Is there anything else that you want to share?

### Data analysis

We sorted audio recordings by serial numbers and saved them date-wise at the end of each day. Voice notes were directly uploaded and saved on Microsoft SharePoint Software. We transcribed the audio recordings and translated them into English at the end of the data collection. We removed identifiers from the transcripts. The transcripts were uploaded and analyzed manually and via qualitative data analysis software MAXQDA 2020. We conducted thematic analysis and followed Creswell's six steps for data analysis. First, the researchers read transcripts and reread them many times to get familiar with the data and develop an interpretation of participants' perspectives of challenges faced during the COVID-19 pandemic. Then, we followed an iterative process of generating codes and grouping the codes together to generate emergent sub-themes. Codes were then labeled, shortened, refined, and analyzed under sub-themes. Finally, we assembled the sub-themes under themes. (NS and DMA) two trained researchers in qualitative research were involved in coding, sub-themes, and themes creation, and discrepancies were resolved with discussion with experienced colleagues (RB and JR) [30, 31].

## Results

The 27 participants (26 nurses and 1 physician) sent in a total of 149 audio recordings. Our analysis identified three themes and eight sub-themes, as shown in Table 1.

**Table 1:** Themes and sub-themes emerging from the data

Themes	Sub-Themes	Evolution of perceptions over time
Individual-level Challenges	Fear of getting infected	The fear of getting infected by COVID was very high in the peak months, and for most of the participants, the fear reduced with time as the COVID cases started to decrease and the HCWs adapted to the new normal
	Feeling demotivated and unappreciated	The HCWs felt emotionally taxed in the initial days of COVID. There was a feeling of sadness as the hospitals could not provide care to all the patients. In addition, they felt demotivated as the patients and the attendants treated them rudely when they could not get beds in the hospital However, with time these negative interactions decreased
	Disappointment due to people’s lack of compliance with COVID-19 protocols	The participants felt disheartened when they saw that the people were taking COVID lightly and were not following COVID-19 precautionary measures (wearing masks and physical distancing). This concern was there even when the number of cases went down in the later months
	Physical Impacts due to heavy PPE use	Almost all the patients found it very difficult to wear the full PPEs. They felt tired, exhausted, and suffocated from wearing PPEs. Some even reported a lack of PPEs initially Over time, the participants still felt it challenging to wear PPEs however they were now used to it. In addition, the participants reported satisfaction with the PPE's availability
Health System Challenges	Infrastructure, logistics, management, and communications response of the hospital	The healthcare workers felt difficulties in the initial months due to smaller, congested areas with reduced space to accommodate increasing cases, limited bed capacity, reduced bipAps/ventilator capacity, difficulty in communication due to heavy PPEs, shortages in the workforce, poor management skills of the workforce for critical patients as the disease was unknown, not trained how to use the PPEs properly The FHCWs expressed relief and appreciation as the hospitals adapted over time by increasing the space and capacity of designated COVID-19 zones, the number of beds, the human resource, provided training in donning and donning of PPEs, improved management skills of critical patients, knowledge of the disease, and timely communication
	Financial stressors	Initially, the participants were apprehensive about how will they make their ends meet if their salaries were reduced The participants expressed much relief in the later months when the salaries were not reduced however there was a disappointment as the overtime salaries were discontinued
Hope for future	Improved disease knowledge and vaccine development trials	In the latter half of the study, the participants felt optimistic about the future and had high hopes for a COVID-free world as there was improved disease knowledge and ongoing trials for vaccine development

### Theme 1: individual-level challenges

The physicians and nurses reported several mental and physical health challenges. These health challenges evolved with time as the COVID-19 pandemic progressed. Commonly occurring sub-themes included fear of getting infected, feeling demotivated and unappreciated, disappointment due to people’s lack of compliance with COVID-19 protocols, and Physical Impacts due to heavy PPE use.

#### Sub-theme 1.1: fear of getting infected

##### *Early perceptions*

As the pandemic reached its peak in June-July, 2020 in Pakistan, most FHCWs were worried and feared that they might get infected while working in the COVID areas of the hospitals. They thought they were at high risk of getting infected as there was an increased patient load, many sick patients arriving at once, patient volume exceeding the bed capacity, and managing patients in congested areas. They were also afraid of infecting their families, loved ones, and friends. Those who had elderly and young children at home were particularly fearful of transmitting infection.

"The main fear I have is that if I catch this infection, I might get healthy again, but if my parents catch it, I might lose them, since they have multiple comorbidities." (WhatsApp Audio 2020–06-04 at 4.15 PM).

##### *Late perceptions*

As the pandemic progressed, in August and September 2020, the fear of infection reduced. Most FHCWs were less anxious as they adapted to the new normal. Some of them said that the pandemic had ended, and COVID-19 did not exist anymore. Others were not worried because they were wearing full PPEs and felt protected. In addition, some believed they were unlikely to get infected as they had a strong faith in God.

However, this fear remained high for some of the workers even during the later stage of the pandemic.

#### Sub-theme 1.2: feeling demotivated and unappreciated

##### *Early perceptions*

During the peak months of June and July 2020, many FHCWs talked about feeling sad and down as the COVID-19 cases increased and the hospitals were unable to provide services to all the patients and had to refuse care to some of the patients. One of the participants said:

"It is an excruciating thing to tell a critical patient that we cannot take you as our beds are full." (WhatsApp Ptt 2020–06-10 at 4.33 PM).

FHCWs felt demotivated when the attendants treated them harshly due to the lack of beds. They felt disrespected and were often verbally abused when patients and families had difficulty obtaining a bed. One of the participants expressed:

“Because we do not have space, we are now getting curses instead of getting good wishes like before COVID days.”

##### *Late perceptions*

The situation seemed to settle towards the end of August, with fewer participants reporting such negative interactions. Few even described receiving verbal appreciation from patients and their families for their hard work. In addition, with time, people reported less anxiety as they adapted to the new normal.

#### Sub-theme 1.3: disappointment due to people’s lack of compliance with COVID-19 protocols

Almost all participants expressed concern over public violations of COVID-19 precautionary measures (wearing masks and social distancing). Participants reported feeling 'disrespected' with all their hard work thrown to waste because the public was not following preventive measures (social distancing, face masks). This augmented the fear of predisposing the FHCWs to infection as well.

“It makes me sad that I am working for people as I am committed to my profession to serve humanity. And they are not even taking care of themselves. We are at risk.” (WhatsApp Pt 2020–06-01 at 8.59 PM).

This concern was reported during the entire duration of data collection, and the trend remained unchanged.

#### Sub-theme 1.4: physical impacts due to heavy PPE use

##### *Early perceptions*

Almost all of the participants reported severe difficulties in wearing PPEs during the hot summer months of Pakistan. The participants described experiencing headaches, dizziness, physical and mental exhaustion, and fatigue. Mostly they used the word “suffocated," particularly about the N95 masks, with many commenting that it was challenging to wear them all day long as it led to breathing difficulties. Many described taking a break “just to breathe” from having worn PPE for an extended period.

"It is challenging to work on COVID patients while wearing PPE. I was profusely sweating and very tired. Moreover, it is challenging to wear the N95 all the time and breathe properly. I was suffocating, but still, I managed to do my duty." (WhatsApp Pt 2020–06-03 at 12.53 PM).

In the initial phase, few of the participants reported a lack of PPEs availability (face shields,

N95 masks, overalls). However, most of the participants reported adequate supplies of PPEs.

##### *Late perceptions*

This concern settled with time as they adapted to work with these SOPs (wearing full PPEs while working in the COVID zones). Towards the end, they reported that even though they still found it challenging to work with the PPEs, they had adjusted to the physical stress of wearing heavy PPE. Despite the physical difficulties, the FHCWs appreciated the hospital administration's adequate and timely provision of PPEs.

### Theme 2: health system challenges

Data demonstrated two sub-themes: Infrastructure, logistics, management, and communications response of the hospital, and financial stressors.

#### Sub-theme 2.1: Infrastructure, logistics, management, and communications response of the hospital

##### *Early perceptions*

The number of patients increased rapidly in June/July '20. Hospitals were converted into (covid/non-covid, hot/cold) zones to accommodate covid patients. This resulted in smaller, congested areas with reduced space. Due to many patients accommodated in limited space, the staff had less space to move around FHCWs felt that the designated COVID-19 zones needed improvements such as; installing proper curtains and railings to reduce infection transmission. FHCWs reported communication difficulties wearing complete personal protective equipment (PPEs). They had to do donning and doffing procedures as they entered and left COVID-19 zones, making it hard to communicate with colleagues, write notes, and give orders. They expressed a need for improving communication strategies in the COVID/non-COVID zones. Some FHCWs talked about short supplies of BiPAP/ventilators hindering patient care and suggested stocking up supplies.

Furthermore, as the number of cases increased, they faced health workforce shortages that fell sick due to COVID-19. This increased the workload burden on the healthcare staff working in the COVID zones due to long shifts and frequent on-call days. Therefore, the participants suggested better management of rosters and scheduling frequent breaks between shifts to avoid burnout among the staff.

"One thing that is making me worried is how frequently we are being assigned here. It would be good if we can get a break and have a rotational schedule." (WhatsApp Ptt 2020–06-01 at 7.32 PM).

##### *Late perceptions*

The FHCWs expressed relief and appreciation as the hospitals adapted over time by increasing the space and capacity of designated COVID-19 zones, the number of beds, the human resource, provided training in donning and donning of PPEs, improved management skills of critical patients, knowledge of the disease, and timely communication. In addition, they felt much safer as they received timely communication from the infectious disease department.

"The other best thing that the department did was develop negative pressure rooms. They made a separate lounge where the COVID staff could take a break. There was no contact between the other staff and COVID staff. That was the best thing." (WhatsApp Audio 2020–06-07 at 7.20. PM).

Another participant said:

"We are getting proper instructions on a timely basis related to the care of COVID-19 suspected or confirmed patients and our safety. We have a separate department in our hospital related to infectious diseases. Moreover, they are responsible for informing us about daily strategies to keep ourselves safe.” (WhatsApp Audio 2020–05-31 at 7.36).

They felt confident in their improved management skills. Finally, they felt happy in saving the lives of critical patients.

“We feel happy to see those patients surviving whom we intubated. That is a personal win for us.” (WhatsApp Audio 2020–08-31 at 10.26 PM).

While some FHCWs were appreciative of the hospital's improved response in accommodating more patients, with an efficient triage system placed for COVID-19 patients, some FHCWs found slow discharge processes resulting in delays and longer length of stay. In addition, this backlog caused difficulty in accommodating newer patients, so the participants felt frustrated when they refused care to critical patients and wanted the hospital administration to have a better management plan instead of constantly diverting patients to other hospitals.

“There are delays in shifting patients which increases the burden in emergency and patient waiting time increases." (WhatsApp Audio 2020–06-01 at 10.52 AM).

#### Sub-theme 2.2: financial stressors

##### *Early perceptions*

As the pandemic progressed and its associated lockdowns were implemented, all felt its economic repercussions. Many participants reported being under stress amidst news of salary reductions. Participants were worried about how will they feed their families and run their houses. In addition, as the overtime pay was discontinued, participants were worried about making their ends meet.

"We are worried that our salaries will be reduced and it will make it difficult for us to run our houses." (WhatsApp Ptt 2020–08-10 at 09.43 PM).

##### *Late perceptions*

The fears and challenges related to salary reductions improved with time. FHCWs expressed relief that they did not have to suffer from a significant financial burden. However, they felt upset as the overtime pay was discontinued.

### Theme 3: hope for future

Research findings demonstrated the sub-theme: Improved disease knowledge and vaccine development trials gave hope for a positive and disease future.

#### Sub-theme 3.1: improved disease knowledge and vaccine development trials

During the later months, many FHCWs felt optimistic about the future as the science progressed and there was improved disease. They felt prepared and better equipped to fight COVID-19 compared to the initial days, which were filled with fear of an unknown disease.

"For this pandemic of COVID-19, we critical care staff working in ED have been taught for the patients proning which is helpful in moderate ARDS patient. So, we can make them not go in that severe ARDS. It was a useful session as we learned about simulation.” (WhatsApp Ptt 2020–08-30 at 8 PM).

Similarly, the FHCWs were hopeful as several research projects were being done, and several vaccines were undergoing trials. Therefore, this was very positive news for some, and they believed that this was the most vital intervention to overcome the disease.

## Discussion

The covid-19 pandemic continues to spread swiftly worldwide. COVID-19 pandemic is a challenge for individuals and the healthcare systems worldwide. Pakistan’s healthcare system is also taken off-guard by the COVID-19 pandemic. Being at the forefront, healthcare professionals have become the most vulnerable. We aimed to document the FHCWs' journey and their perception of the health systems' performance during the peak and trough of the first wave of the COVID-19 pandemic. It is the first qualitative longitudinal study in a real-time pandemic to capture healthcare workers' evolution of perceptions in Pakistan.

There were several mental and physical health challenges expressed by the FHCWs as they worked on the frontline during the COVID-19 pandemic. In the initial days, the fear of getting infected and transmitting the infection to loved ones was extremely high. They believed they were at high risk because of unprecedented patient load, less bed capacity, lack of understanding of the disease, and uncertainty about patient outcomes. Similar concerns have been expressed in studies from countries such as the People's Republic of China, Islamic Republic of Iran, Lebanon, Brazil, and Pakistan in expressed high fear of getting infected due to their jobs [32–36]. FHCWs expressed helplessness as the hospitals reached maximum capacity, and patients had to be turned away. Many FHCWs felt physical exhaustion, fatigue, tiredness, headaches, dizziness, and suffocation due to wearing PPEs.

However, there was a positive and improved change in the perceptions of FHCWs in the later months. The initial fear of getting infected and transmitting disease reduced over time as they adapted to the new normal. They felt protected by using PPEs. The FHCWs felt physical impacts (exhaustion, fatigue, tiredness), but with time they got used to wearing the PPEs. Due to patients being turned away from hospitals due to capacity issues, the anxiety also settled with time. However, FCHWs felt disappointed because the general public's non-compliance with precautionary measures (wearing masks and social distancing) was reported throughout the study.

Health systems worldwide were compromised in the face of the deadly pandemic. Pakistan also faced tremendous challenges. During the initial/peak period, there was a lack of space and high patient volumes, and hospitals were put on diversion due to lack of space, difficulties in managing critical patients due to limited knowledge, difficulty in communication due to full PPE gear, shortage of human resource and lack of BiPAP/ventilators. Nearly most of the countries reported similar challenges [7, 37].

During the later months, the FHCWs expressed relief as the hospitals adapted over time by increasing the number of beds, the human resource, capacity building, providing training in donning and donning of PPEs, improved management skills of FCWS critical patients, improved knowledge of the disease, and timely communication.

FHCWs shared several positive experiences. First, they took pride and expressed satisfaction in saving lives. In addition, the participants felt optimistic about the future outlook; the numbers had gone down significantly, improved disease knowledge and management, and vaccine trials were looking good.

Meaningful learning from this study is that WhatsApp was an easy-to-use tool that generated a large amount of rich data in a relatively short interval. Moreover, since the data was self-generated, the resources required for data collection were limited.

### Study limitations

First, we found that the number of self-recorded audios decreased as the study progressed. The participants were asked why; they reported feeling tired due to heavy workload during their shifts and were too tired to take on an additional task. For these participants, we offered a telephonic interview over weekends and at the time of their convenience. Secondly, study participants felt they did not have any particularly new data to report after each shift. We addressed this issue by changing the reporting frequency from each shift to reporting once a week. Thirdly, our study participants were from private sector tertiary care hospital EDs. Unfortunately, despite several attempts, we could not get FHCWs from the public sector to participate in the study. The perception of public sector FHCWs might be different as resources are often limited, and the workload is high in these settings. Finally, after four months, we had to stop the data collection process as the number of COVID-19 patients decreased, and the participants felt they had nothing new to report.

## Conclusion

This longitudinal study outlines several lessons learned first-hand during the pandemic. The first lesson was that the individuals and systems were not prepared to deal with a calamity of this scope. The healthcare workers felt taxed and overwhelmed emotionally, mentally, and physically. Second, the systems struggled to cope with increased cases because of weak infrastructure, less hospital and bed capacity, human resource shortages, lack of capacity of healthcare workers, and frail administrative and management measures. Thirdly, the systems and individuals responded with zeal, rigor, and bravery to deal with the COVID situation in Pakistan. The hospitals increased the bed capacity, improved healthcare workers' capacity via training, invested in providing supplies (PPEs, BiPAP/ventilators), ensuring timely information, and improved communication. This study provides essential information to make important policy decisions to better equip the systems and individuals for future pandemic readiness.

## Data Availability

The data supporting this study's findings are available from the corresponding author upon reasonable request.

## Abbreviations

COVID-19: Coronavirus disease
FHCWs: Frontline Healthcare Workers
PPE: Personal Protective Equipment
LMICs: Low- and middle-income countries
PSEM: Pakistan Society of Emergency Medicine
ED: Emergency Department
